# The Variability of Neural Responses to Naturalistic Videos Change with Age and Sex

**DOI:** 10.1523/ENEURO.0244-17.2017

**Published:** 2018-01-27

**Authors:** Agustin Petroni, Samantha S. Cohen, Lei Ai, Nicolas Langer, Simon Henin, Tamara Vanderwal, Michael P. Milham, Lucas C. Parra

**Affiliations:** 1 Department of Biomedical Engineering, City College of New York, New York, NY 10031; 2 Department of Psychology, the Graduate Center of the City University of New York, New York, NY 10016; 3 Center for the Developing Brain, Child Mind Institute, New York, NY 10022; 4 Methods of Plasticity Research, Department of Psychology, University of Zurich, 8050, Switzerland; 5 Yale Child Study Center, New Haven, CT 06520; 6 Nathan Kline Institute for Psychiatric Research, Orangeburg, NY 10962

**Keywords:** Development, EEG, evoked responses, inter-subject correlation, naturalistic stimuli

## Abstract

Neural development is generally marked by an increase in the efficiency and diversity of neural processes. In a large sample (*n* = 114) of human children and adults with ages ranging from 5 to 44 yr, we investigated the neural responses to naturalistic video stimuli. Videos from both real-life classroom settings and Hollywood feature films were used to probe different aspects of attention and engagement. For all stimuli, older ages were marked by more variable neural responses. Variability was assessed by the intersubject correlation of evoked electroencephalographic responses. Young males also had less-variable responses than young females. These results were replicated in an independent cohort (*n* = 303). When interpreted in the context of neural maturation, we conclude that neural function becomes more variable with maturity, at least during the passive viewing of real-world stimuli.

## Significance Statement

Naturalistic videos were used to probe response variability with EEG in a large developmental cohort. Our results are consistent with developmental theories positing that neural variability increases with maturation, and that neural maturation typically occurs earlier in females. These results differ from those observed with fMRI, where an increase in stereotyped responses with age is observed during development.

## Introduction

This study examines the relationship between the variability of neural responses and development. Over the course of development, the accuracy and stability of behaviors generally increase. This performance improvement is typically accompanied by a seemingly paradoxical increase in the variability of neural responses both within and across subjects (Grady, 2012; Dinstein et al., 2015). More-variable electroencephalographic (EEG) responses across trials, characterized by an increase in dimensionality and entropy, are associated with lower reaction time variability and higher recognition accuracy (Mcintosh et al., 2008). Neural variability often presents as an increase in the complexity of neural responses. This may be due in part to a developmental increase in the repertoire of possible brain states (Vakorin et al., 2013), and this increase in complexity may underlie the integration between distributed neural populations (Vakorin et al., 2011). EEG signal complexity becomes elevated in late adolescence and is also elevated in females relative to males at this stage, indicating that females may attain mature brain functioning before males (Anokhin et al., 2000). Anatomic studies generally support the notion that females reach neural maturity before males (Giedd et al., 1999; Lenroot and Giedd, 2006; Lenroot et al., 2007; Marsh et al., 2008).

Neural variability does not always accompany proficient behavior, however. Both theta band coherence, a performance-monitoring measure, and behavior are more variable across trials in children (Papenberg et al., 2013). This suggests that neural variability does not always increase with maturation. For adults, the variability in “functional connectivity” between different networks measured with functional MRI (fMRI) is elevated during rest and decreases during a cognitive task. The reverse is true for children, whose brains become more variable during the task, and their performance is expectedly lower than adults (Hutchison and Morton, 2015).

Recently, responses to naturalistic narrative stimuli have been used to examine how variability in behavior and neural activity change with development. In these cases, variability is measured across subjects rather than within individuals because it is expected that if an individual has a less-variable neural response across repeated renditions of stimulus, their neural response will also be more similar to others who are responding to the same stimulus. Adults watch *Sesame Street* more similarly to each other than infants do, as assessed by where their eyes fixate (Kirkorian et al., 2012). Additionally, adults have more broadly similar neural responses to *Sesame Street* than children (Cantlon and Li, 2013). Although the neural responses of adults to *Sesame Street* correlate more with each other in many parietal and frontal regions, children correlate more strongly with each other in a specific region in the superior temporal cortex (Cantlon and Li, 2013). Generally, from ages 18 to 88 yr, as humans age, responses to videos increase in variability (Campbell et al., 2015). Taken together, these studies demonstrate that neural variability changes with age. The nature of this relationship depends on multiple factors including the metric of neural variability, the developmental stage sampled, and the brain regions of interest.

Here, EEG was recorded from subjects with ages ranging from 5 to 44 yr as they were presented with both naturalistic (Dmochowski et al., 2012, 2014) and conventional stimuli. To assess neural variability, the level of similarity across subjects was assessed with the intersubject correlation (ISC) of responses evoked by the stimuli. ISC of the EEG is indicative of attention, engagement, and memory in healthy adults (Dmochowski et al., 2014; Cohen and Parra, 2016; Ki et al., 2016; Cohen et al., 2017). We found that neural responses, indexed by ISC, become more variable with age. Among children, females have more-variable neural responses than males. This increase in variability is not due to a decrease in evoked response magnitude and was reproduced in two independent cohorts consisting of 114 and 303 individuals. These results are consistent with theories positing that development coincides with an increased repertoire of neural representations (Mcintosh et al., 2008), and the sex differences are consistent with the idea that young males are less neurally mature than young females (Giedd et al., 1999; Lenroot and Giedd, 2006; Lenroot et al., 2007; Marsh et al., 2008). Importantly, this is the first EEG study to report a measure of across-subject neural similarity with clear age and sex effects.

## Materials and Methods

### Subjects

**Figure 1.**
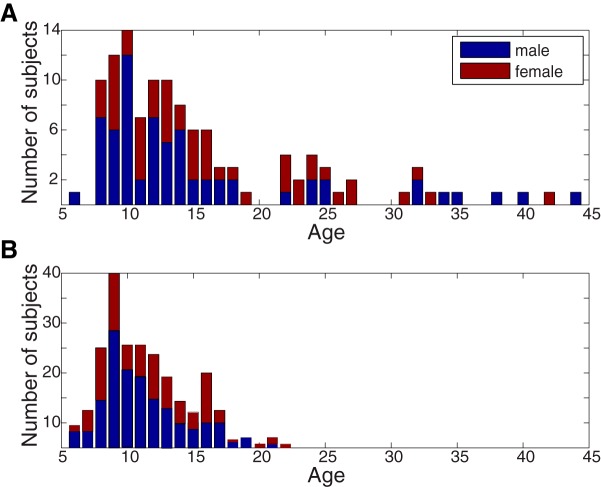
Age and sex distributions for the main study (***A***) and replication study (***B***). ***A***, Subjects in the main study (*n* = 114) had data for all of the stimuli. ***B***, Subjects in the replication study (*n* = 303) had only three stimuli (Wimpy, Fract, DesMe) and contribute to the results in Fig. 9.

In the main study, ages ranged from 6 to 44 yr (*n* = 114, 14.2 ± 8.0 yr, 46 females; see Fig. 1*A* for a full age and sex distribution) as part of the Child Mind Institute–Multimodal Resource for Studying Information Processing in the Developing Brain (http://fcon_1000.projects.nitrc.org/indi/cmi_eeg/; Langer et al., 2017). In the replication study, ages ranged from 5 to 21 yr (*n* = 303, 11.3 ± 3.9 yr, 135 females; see Fig. 1*B* for a full age and sex distribution). These data were obtained from the Child Mind Institute Healthy Brain Network (http://fcon_1000.projects.nitrc.org/indi/cmi_healthy_brain_network/; Alexander et al., 2017). Both the main and replication study data come from publically available datasets. All experiments were performed in accordance with relevant guidelines and regulations. The study was reviewed and approved by the Chesapeake Institutional Review Board. All subjects presented with normal or corrected-to-normal vision.

### Stimuli

Engaging, naturalistic videos were the primary stimuli. Specific videos were selected because they contained content relevant to social cognition, classroom anxiety, and attention. Three videos either featured educational content or depicted classroom scenarios: *Fun with Fractals* (Fract, MIT), a cartoon that explains fractals with examples (4 min 34 s), *How to Improve at Simple Arithmetic* (Arith, E-How), in which a math teacher in a typical educational setting explains addition and multiplication (1 min 30 s), and *Pre-Algebra Class* (StudT, Pearson Education), showing an interaction between two students and a teacher (StudT, for student–teacher interaction) during math problem solving (1 min 40 s). Two videos were clips from conventional cinema: *Diary of a Wimpy Kid* (Wimpy, Universal Pictures), a movie about a preteen starting middle school (1 min 57 s), and *Despicable Me* (DesMe, Universal Pictures), which contains infant and toddler characters and emphasizes social interactions (2 min 51 s). Although the main cohort contains data from all stimuli, the replication cohort only had three stimuli: Wimpy, Fract, and DesMe. The variability of the neural responses to these videos was measured across subjects using the ISC of evoked responses (see below). As a control condition, a “Rest” condition, during which subjects sat with their eyes closed for 4 min 20 s, was also analyzed. This period establishes the baseline level of ISC, as no time-aligned stimulus entraining neural activity across subjects was presented. Finally, Flash, a stimulus condition without any narrative content, was used. During this stimulus, a black-and-white grating pattern that flashed at 25 Hz was presented for three minutes, thus synchronously stimulating neural activity across subjects (see Steady-state visual evoked potentials Methods section). This stimulus elicits steady-state evoked potentials (Vanegas et al., 2015) and was included to explore the extent to which ISC is driven by low-level evoked responses.

### Procedure

While seated in a dimly lit room wearing an EEG net, subjects watched a series of short videos in a pseudorandom order. Stimuli were presented on a 17-inch CRT monitor (SONY Trinitron Multiscan G220, display dimensions 330 × 240 mm, resolution 800 × 600 pixels, vertical refresh rate of 100 Hz). Note that some subjects did not experience all stimuli due to time limitations (Langer et al., 2017). Additionally, as explained below, poor data quality for some recordings caused additional data loss. For the replication study, only three conditions were used: Wimpy, Fract, and DesMe.

### EEG recordings and preprocessing

EEG recordings were performed with an EGI Clinical Geodesic 128-channel system (Electrical Geodesic). Of the 128 channels recorded, 105 constituted the EEG recording and 11 represented electro-oculography (EOG) channels used for eye movement artifact removal. The remaining channels, mainly recording from the neck and face, were discarded. First, noisy channels were selected by visual inspection and replaced with by zero-valued samples, thus eliminating those channels’ contribution in subsequent calculations of covariance matrices. Recordings, initially at 500 Hz, were then downsampled to 125 Hz, high-pass–filtered at 1 Hz, and notch-filtered between 59 and 61 Hz with a fourth-order Butterworth filter. Eye artifacts were removed by linearly regressing the EOG channels from the scalp EEG channels (Parra et al., 2005). Next, a robust principal components analysis (PCA) algorithm, the inexact augmented Lagrange multipliers methodp (Lin et al., 2013), was used to remove sparse outliers from the data following Ki et al. (2016). Briefly, robust PCA recovers a low-rank matrix, **A**, from a corrupted data matrix **D** = **A** + **E**, where some entries of the additive errors **E** may be arbitrarily large. Finally, individual recordings for some stimuli were discarded on the basis of visual inspection because they remained noisy after both automatic and manual noise removal. This was necessary because these subjects exhibited profound movement artifacts or the saline used for the recordings dried out. Despite these steps taken, the data overall appear to be of poorer quality than that collected in an electrically shielded room with conductive gel (saline was used here). The noise in the data may have led to the relatively low ISC values reported compared with previous studies (Ki et al., 2016; Cohen et al., 2017). However, it is unlikely that the noise contributed to our results, as under baseline conditions (Rest), there was no difference in power between the cohorts (see Results). All signal processing was performed offline using Matlab software (MathWorks).

### Intersubject correlation

As variability is the inverse of similarity, we measured the similarity of evoked EEG responses across subjects. This approach has been used extensively to study concerted, intersubject changes in blood-oxygen level–dependent (BOLD) signal in fMRI (Hasson et al., 2004, 2009; Kauppi et al., 2010) and has been adapted to leverage the improved time resolution facilitated by EEG. To determine the neural similarity across subjects responding to the same stimulus (or in the same condition, in the case of Rest) the ISC of the EEG signal was computed as described previously (Dmochowski et al., 2012, 2014; Cohen and Parra, 2016; Ki et al., 2016). ISC assesses the level of correlation in the EEG across time among a group of subjects as they respond to the same stimulus. Larger ISC values imply more similarity in fast EEG responses across subjects (<1 s). This indicates that the signals are more reliable due to decreased intersubject variability. It has also been found that subjects who pay more attention to the stimulus have higher ISC values (Ki et al., 2016). An advantage of the technique is that the stimulus need only be presented once to each subject, because evoked responses are compared across individuals. As repeated trials are unnecessary, responses are more similar to natural situations in which people experience uniquely presented novel stimuli. Additionally, in contrast to event-related potentials (ERPs), the technique can be applied to continuous and dynamic natural stimuli without the need for specific event markers (Ben-Yakov et al., 2012). As such, the approach is data driven both spatially and temporally—spatially, because the data from the subjects determines the best combination of electrodes (which are spatially distributed across the scalp and therefore may correspond with different anatomic regions) that maximize the correlation across subjects, and temporally, because the ultimate correlation values are determined by the temporal fluctuations in the EEG signals. In contrast, a more traditional approach to EEG data analysis would be to select electrodes that have previously been shown to elicit a certain effect (or ERP) and measure event-locked responses from these electrodes. We are not taking this approach. Rather, the electrodes that we chose and the time periods that maximize correlation are determined directly by the data itself.

ISC utilizes correlated component analysis to identify linear combinations of EEG electrodes that capture most of the correlation across subjects (Dmochowski et al., 2012). Correlated component analysis is similar to PCA except that rather than maximizing variance within one dataset, it selects projections, $v\;\in \;R^{D}$, where *D* is the number of electrodes, that maximize the correlation between multiple datasets. These projections can be thought of as virtual sensors (or component sources) of activity that are optimized to capture most of the correlation between subjects. They are the eigenvectors of $R_{W}^{-1}R_{B}$, where $R_{W}$ is the average within-subject covariance, $\frac{1}{N}\sum_{k}R_{kk}$, and $R_{B}$ is the average between subjects cross-covariance, $\frac{1}{N\left(N-1\right)}\sum_{k}\sum_{l,l\neq k}R_{kl}$, and $R_{kl}=\sum_{t}\left[x_{k}\left(t\right)-{\bar{x}}_{k}\right]{\left[x_{l}\left(t\right)-{\bar{x}}_{l}\right]}^{T}$ measures the cross-covariance of all electrodes in subject *k* with all electrodes in subject *l*. Vector $x_{k}\left(t\right)$ is the scalp voltages at time *t* in subject *k*, and ${\bar{x}}_{k}$ is their mean value in time.

Following previous research, we use the three components, or eigenvalues of $R_{W}^{-1}R_{B}$, that represent the largest fraction of the correlation across subjects. These components can be optimized for all subjects together, or for a subset of the entire cohort. The subsets used in this paper are stimulus (Wimpy, DesMe, Fract, Arith, StudT, Flash, and Rest), age group (young vs. old), sex (male vs. female), and sex and age group combined (young-male, young-female, old-male, and old-female). ISC components are computed within subsets of the entire sample to examine potential differences in the spatial distribution of activity across groups, although the spatial patterns are largely consistent (Fig. 7).

To calculate the ISC for individual subjects as they respond to the same condition as their peers, the correlation between each individual’s EEG responses and the responses from all other individuals is calculated (Cohen and Parra, 2016; Ki et al., 2016). The ISC values reported throughout the paper are this measure of how well each individual correlates with the others. The projections, $v\;\in \;R^{D}$, used to compute this subject-specific ISC value are computed either across all subjects or within the subgroups listed above (divided by either stimulus, age, sex, or age + sex). The ISC for each subject is therefore

$$C_{ik}=\frac{v_{i}^{T}R_{b,k}v_{i}}{v_{i}^{T}R_{w,k}v_{i}},$$

where $R_{b,k}=\frac{1}{\left(N-1\right)}\sum_{l,l\neq k}\left(R_{kl}+R_{lk}\right)$ and $R_{w,k}=\frac{1}{\left(N-1\right)}\sum_{l,l\neq k}\left(R_{kk}+R_{ll}\right)$. ISC for subject *k* is therefore $\sum_{i=1}^{3}C_{ik}$. A simplified template for the code to compute the correlated components and the ISC for individual subjects is available at http://www.parralab.org/isc/

### Steady-state visual evoked potentials

To determine the strength of low-level sensory evoked responses across individuals, we leveraged the steady state visual evoked potential (SSVEP) paradigm (Flash) that was part of the data collection effort (Langer et al., 2017). Stimulus and analysis followed established techniques (Vanegas et al., 2015). Briefly, the stimulus consisted of four circular “foreground” stimuli (vertical grating, radius 2°) that were flickered on and off at 25 Hz and embedded in a static “background” grating, which is known to generate reliable SSVEPs (Vanegas et al., 2015). This stimulus was presented in trials of 2.4-s duration with intertrial intervals of 1 s, which included a fixation cross presented for 0.5 s. The stimuli were presented in several conditions that varied in their contrast and in the phase relationship between the foreground and the background. A total of 128 trials were present (12 conditions total: four foreground contrasts: 0%, 30%, 60%, and 100%, and three background conditions: parallel phase, orthogonal phase, and no surround stimuli). Artifacts were rejected by removing trials for which the power (or absolute value) of any electrode exceeded more than three standard deviations above the mean. EOG activity was regressed out of the EEG, as described above. The initial 200 ms of each trial was removed to eliminate the onset of the visual evoked response. Data were Fourier transformed for each trial, and power in a 0.5-Hz bin surrounding the 25-Hz band was extracted and then averaged across all trials, regardless of condition (thus ignoring details of the foreground–background interaction). Because the EEG activity measured with this paradigm is known to be dominated by primary visual cortex (V1) responses, power was averaged over the five most relevant occipital electrodes (O1–O5; (Vanegas et al., 2015).

### Dimensionality of EEG responses

To gain a sense of the dimensionality of the EEG responses across subjects, the eigenvalue spectrum was extracted from each subject’s covariance matrix (covariance between all electrodes measured across time). These covariance matrices measure the correlation between electrodes for each subject. The sum of the eigenvalues represents the overall power in the data. To assess the dimensionality of the data, lines were fitted to the log-log plot of the eigenvalue spectrum of each subject’s covariance matrix. A shallower slope of the linear fit indicates that the there is appreciable power over a larger number of dimensions. Two-way ANOVAs and subsequent *post hoc t* tests were employed to compare power and the slopes of these linear fits for each age and sex group both across all stimuli and within each stimulus.

## Results

We sought to determine whether and how the variability of EEG differs across age and sex in children and adults ranging from 6 to 44 yr of age. To assess the variability in EEG signals across subjects, the ISC between individuals and their peers was assessed in response to both naturalistic videos and artificial stimuli. ISC can be thought of as a measure of the similarity of neural responses (Dmochowski et al., 2012). If subjects respond more similarly to their peers, they will have a larger ISC value, which indicates that they have a less-variable neural response.

### ISC varies between stimuli

ISC is a stimulus-driven measure of attention (Ki et al., 2016), because neural responses are more correlated across subjects when they naturally attend to a stimulus than when they are engaged in a dual task. It is therefore expected to be indicative of varying levels of engagement (Cohen et al., 2017). A one-way ANOVA determined that ISC significantly depended on the stimulus (*F*(7) = 78.26, *p* = 10^−68^; mean ± STD ISC values: Wimpy: 0.053 ± 0.036; DesMe: 0.035 ± 0.023; Arith: 0.019 ± 0.013; Fract: 0.026 ± 0.016; StudT: 0.012 ± 0.009; Flash: 0.030 ± 0.019; Rest: 0.001 ± 0.004), indicating that the stimuli significantly varied in engagement level (Cohen et al., 2017). It is worth noting that these ISC values are relatively low compared with previous research (Cohen and Parra, 2016; Ki et al., 2016). There are two factors that contribute to this discrepancy: the lower production quality and therefore engagement level elicited by these stimuli and the relatively poor quality of the EEG data (see Methods). Note also that ISC for EEG is generally lower than ISC of fMRI (e.g. Lahnakoski et al., 2017), which has a slower time scale and higher signal-to-noise ratio, factors that can both contribute to higher correlations (Haufe et al., 2017). As expected, ISC in the Rest condition was not significantly different from zero (*t* test, *t*(45) = 0.52, *p* = 0.4), confirming the notion that ISC reflects stimulus-induced correlations (Dmochowski et al., 2012). A one-way ANOVA was therefore performed on all stimuli excluding Rest, confirming that ISC strongly varies between stimuli (*F*(6) = 71.70, *p* = 10^−55^). Tukey *post hoc* pairwise comparisons revealed that ISC was significantly stronger when evoked by the qualitatively more engaging stimuli (Wimpy and DesMe), than it was for educational videos (Arith, Fract, StudT; Tukey *post hoc* pairwise comparisons between each pair of videos, Tukey’s HSD: *p* < 10^−4^). Among the more engaging videos from conventional cinema, Wimpy, a movie trailer for the feature film “Diary of a Wimpy Kid,” evoked a higher level of neural similarity than DesMe, a scene from the animated film “Despicable Me” (Tukey’s HSD: *p* = 10^−7^). Among the relatively less-engaging educational videos, Fract elicited the highest level of ISC, which was significantly higher than StudT (Tukey’s HSD: *p* = 10^−6^), but not Arith (Tukey’s HSD: *p* = 0.2). Interestingly, Arith elicited a level of ISC similar to Flash (Tukey’s HSD: *p* = 0.5), and the level of ISC elicited by Flash was significantly higher than StudT (Tukey’s HSD: *p* = 10^−7^).

### ISC decreases with age

**Figure 2.**
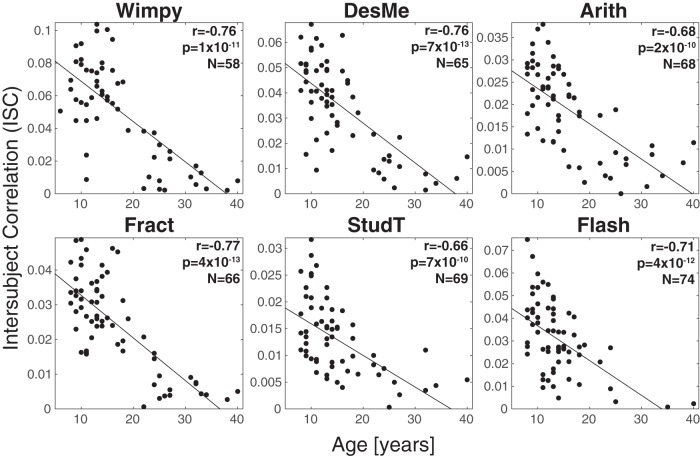
Neural similarity, measured as the ISC of neural activity, decreased with age. Correlation values ranged from *r* = –0.58 to *r* = –0.78, indicating a consistent relationship between maturity and neural variability. ISC was computed for each individual by correlating neural responses from individual subjects with the neural responses from all other subjects for that stimulus (regardless of age and sex).

We hypothesized that neural similarity changes with age and therefore examined the correlation between ISC and age. Here, ISC is computed in individuals by measuring the extent to which each subject correlated with the other people in the same stimulus condition. For all of the stimuli excluding Rest, there was a negative relationship between age and ISC (all *r* = –0.68 ± 0.09, all *p* < 10^−10^, false discovery rate corrected following Benjamini and Hochberg (1995) (Fig. 2). ISC did not vary with age during Rest (*r* = –0.10, *p* = 0.5, *n* = 46). This was expected, since Rest contained no stimulus to drive EEG signal similarly across subjects.

**Figure 3.**
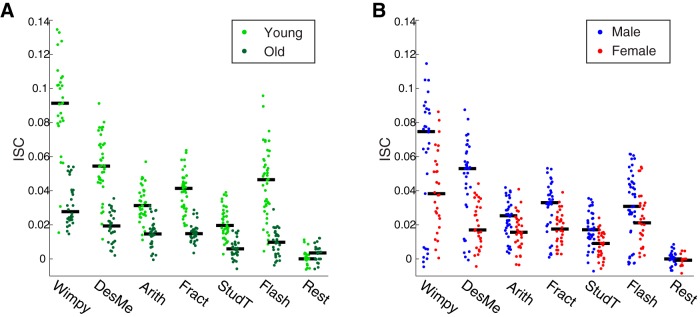
ISC, a measure of neural similarity, was consistently higher among younger ages and males. ***A***, Across all stimuli, ISC was higher for younger subjects (6–14 yr, light green) than older subjects (15–44 yr, dark green). ***B***, Across all stimuli, ISC was higher for males (blue) than females (red). For both ***A*** and ***B***, ISC was computed separately within each age and sex group. Black lines indicate the median.

These results indicate that ISC decreases with age. However, most of the subjects in the main study were from the lower half of the age distribution (Fig. 1*A*). Because the components used to measure ISC are optimized to capture the correlation across all subjects, the components may have been biased by these younger subjects, who constituted a majority of the sample. The cohort was therefore divided into two age groups of equal size to eliminate this potential measurement bias. The median split resulted in groups whose ages ranged from 6 to 14 (mean age 10.74 ± 2.03 yr) and 15 to 44 (mean age 23.65 ± 8.04 yr). The ISC was then recomputed from components extracted separately in each group. A two-way ANOVA with factors of age and stimulus revealed that ISC was significantly modulated by both stimulus (all excluding Rest, *F*(5, 393) = 63.64, *p* = 10^−47^) and age (*F*(1, 393) = 335.46, *p* = 10^−53^; Fig. 3*A*). For all stimuli, ISC was much higher in the younger age group.

### ISC is elevated in males

**Figure 4.**
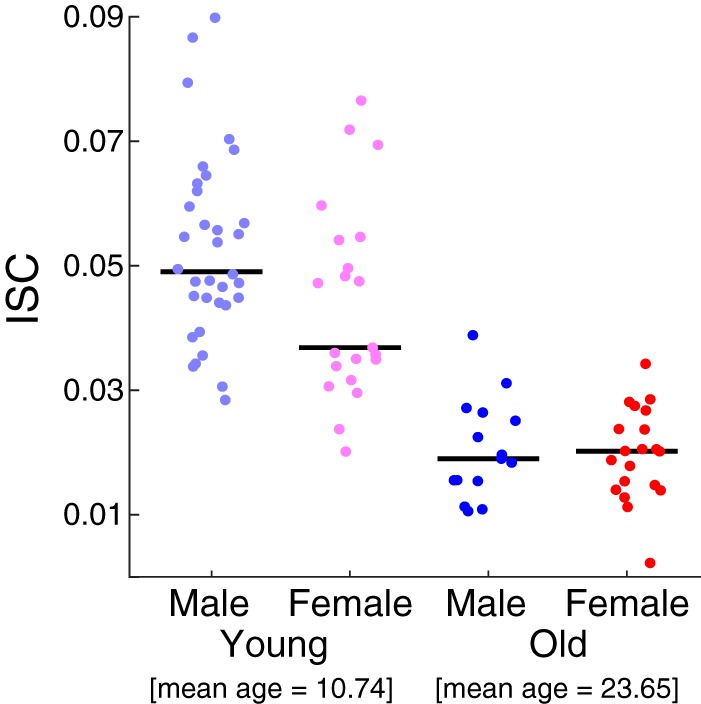
Sex differences in the young do not exist in the old. Young males were more neurally similar to each other than young females. This sex difference is absent in the older group. Here, ISC was computed within each sex and age group separately and averaged across all stimuli except for Flash and Rest. Black lines indicate the median.

Sex is an important factor that influences the developmental trajectory of the human brain (Giedd et al., 1999; Lenroot and Giedd, 2006; Lenroot et al., 2007; Marsh et al., 2008). We therefore explored the relationship between sex and ISC. A two-way ANOVA with factors of sex and stimulus (excluding Rest) revealed main effects for both sex (*F*(1, 393) = 53.11, *p* = 10^−12^) and stimulus (*F*(5, 393) = 30.12, *p* = 10^−26^; Fig. 3*B*). Tukey’s *post hoc* tests revealed that ISC was consistently higher in males for all stimuli except for Flash, where it was marginally significant (Flash: *p* = 0.06; Wimpy: *p* = 0.03; DesMe: *p* = 10^−6^; Arith: *p* = 0.003; Fract: *p* = 10^−4^; StudT: *p* = 10^−4^). To examine whether the sex difference depended on age, the data were separated into four groups with the same age division between 14 and 15 yr as above (young-male, young-female, old-male, old-female). ISC was measured within each group and averaged across all stimuli available for each subject to ensure sufficiently large sample sizes (excluding control conditions, Flash and Rest; Fig. 4). A two-way ANOVA with sex and age as factors confirmed the age effect (*F*(1,87) = 98.85, *p* = 10^−16^), and the sex effect was marginally significant (*F*(1,87) = 3.83, *p* = 0.05). A direct comparison between the sexes in each age group revealed that the sex effect was marginally significant among the young ages (*t*(53) = 2.02, *p* = 0.05, 6–14 yr), but not present for the old ages (*t*(33) = 0.28, *p* = 0.8, 15–44 yr).

### The effect of age on ISC is not due to evoked response difference

**Figure 5.**
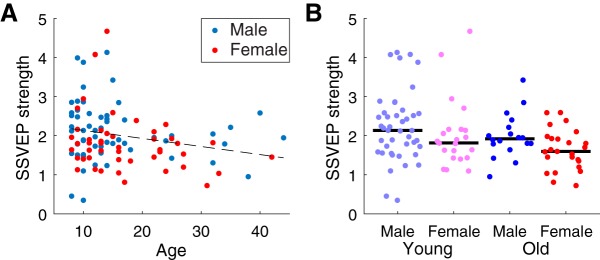
SSVEP magnitude depended on age, but not on sex. ***A***, SSVEP strength was weakly correlated with age across subjects, but it was no different between males and females. ***B***, SSVEP strength was no different between males and females. Black lines indicate the median.

The relationships between ISC, age, and sex may be partially driven by the reduction of evoked response magnitude with age (Goodin et al., 1978; Tomé et al., 2015). Although correlation, which ISC measures, is theoretically independent of magnitude, it is possible that a decrease in magnitude corresponds with a decrease in the signal-to-noise ratio, which would result in a smaller ISC. The magnitude of evoked responses was therefore assessed with the Flash stimulus, which elicited SSVEPs (see Methods). SSVEP magnitude weakly declines with age (*r* = –0.22, *p* = 0.02, *n* = 109; Fig. 5*A*) and a two-way ANOVA with age and sex as factors (the same age/sex groups as Fig. 4) found the age effect to be marginally significant (*F*(1106) = 4.00, *p* = 0.05; Fig. 5*B*). There was no significant relationship between sex and SSVEP strength (*F*(1106) = 3.3, *p* = 0.08).

**Figure 6.**
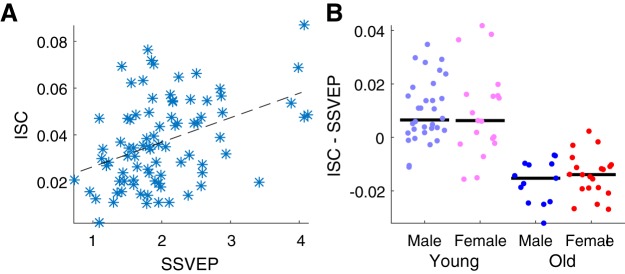
Relationship between ISC magnitude and SSVEP strength. ***A***, SSVEP strength, a measure of the magnitude of evoked responses, correlated with ISC strength, calculated using all stimuli except for Flash and Rest. ***B***, Comparison of ISC strength after SSVEP magnitude was regressed out (ISC – SSVEP) between males and females in the young and old age groups. Although there was a significant difference between the age groups, a difference between the sex groups was not present. Black line indicates the median.

Because both SSVEP amplitude and ISC decrease with age, we reasoned that SSVEPs could be used to factor out the effect of evoked response strength (Goodin et al., 1978; Tomé et al., 2015). Indeed, ISC and SSVEP amplitude are correlated across subjects (*r* = 0.41, *p* = 0.0001, *n* = 84; Fig. 6*A*). To control for the effect of evoked response strength, each individual’s SSVEP amplitude was linearly regressed against ISC, and the portion that could be explained by the SSVEP was subtracted (ISC calculated within the same age/sex group as Fig. 4). A two-way ANOVA with age and sex as factors revealed that this residual ISC still significantly varies with age (*F*(1,81) = 85.49, *p* = 10^−14^), but does not vary with sex (*F*(1,81) = 0.08, *p* = 0.8; Fig. 6*B*). Additionally, the sex effect is no longer present in the younger group when SSVEP strength is controlled for (*t*(49) = 0.11, *p* = 0.9). The lack of a sex effect may mean that the relationship between sex and neural variability is due in part to evoked response magnitude, but the lack of an effect may also result from the reduced number of subjects for which SSVEP magnitude was available: 84 versus 114. Regardless, neural variability, as assessed by ISC, does increase with age, regardless of the strength of evoked responses.

### Correlated component topographies are similar across age and sex groups

**Figure 7.**
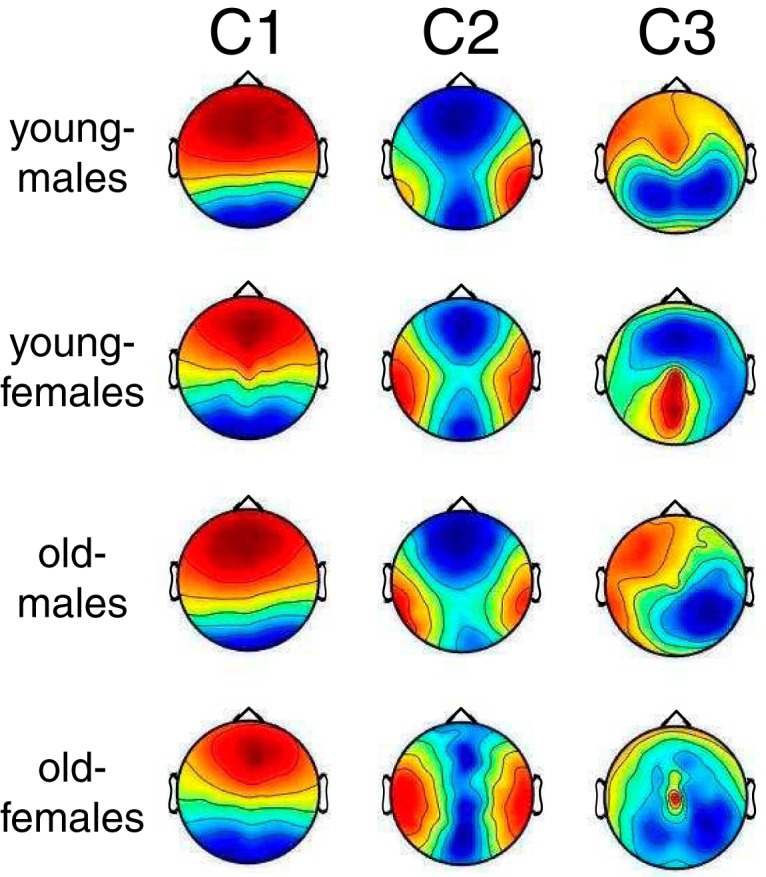
Spatial distributions corresponding to the three strongest components of ISC (C1–C3). Red and blue colors indicate positive and negative correlation of the voltages on the scalp surface with the component activity. These maps are unitless due to an arbitrary scale on the projection vectors. Here, the projections have been computed separately for the combination of the two sex and age groups. As the scalp topographies were relatively consistent across the groups, the differences in ISC across these groups were not due to differences in the spatial topography of correlation within the group.

ISC was measured using components of the EEG that maximize correlations between subjects. These components are linear combinations of electrodes and can be thought of as virtual sensors (see Methods). To determine whether the spatial distribution of the corresponding activity differed across groups, the “forward” model, which represents how the components look on the surface of the scalp, was computed for the largest three components that were used to compute ISC (Parra et al., 2005). These component topographies were very similar across all age/sex groups for the strongest two components, C1 and C2 (Fig. 7, minimum cosine similarity 0.97 for C1 and 0.78 for C2). The third component (C3) was less similar across the groups (cosine similarity 0.89–0.31), but it also constituted a much weaker portion of the ISC (C1 = 0.016 ± 0.009, C2 = 0.008 ±0.005, and C3 = 0.004 ± 0.003, computed as in Fig. 4 and averaged across all subjects and stimuli). Thus, for the most part, differences in ISC between age and sex groups were not due to differences in the spatial distribution of neural activity across these groups.

### Dimensionality of EEG responses

**Figure 8.**
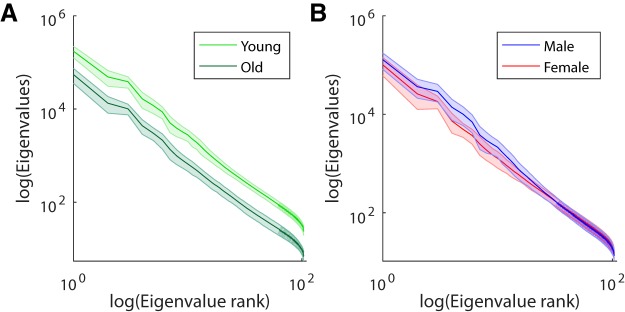
Eigenvalue spectra of the average covariances for each demographic group. Eigenvalues measure the power of the signal in principal components of the EEG (correlated across time for each stimulus). Each curve is the average eigenvalue spectrum for each group averaged across all stimuli and subjects. ***A***, Young subjects have more power than old subjects in all dimensions. This is represented by the upward shift in their average eigenvalue spectrum. ***B***, The eigenvalue spectrum of females has a shallower slope than that for males, indicating that they have a more diverse set of neural responses.

To determine whether the differences in ISC across groups was due to diverse responses across subjects or to more highly dimensional responses within subjects, the eigenvalue spectra of the EEG covariance matrices were analyzed (Fig. 8). The sum of these spectra represents the overall power of the data. In general, the younger age group (using the same median split as above) had more power than the older age group across all stimuli (*F*(1438) = 452.13, *p* = 10^−69^). This suggests that there was more overall power in the EEG of the young group. This power difference was present only during the stimuli, not during rest (*t*(44) = 0.6, *p* = 0.5), suggesting that younger subjects have stronger stimulus-driven evoked responses. To assess the dimensionality of the EEG responses, a linear model was fitted to each subject’s eigenvalue spectrum (see Methods), and the slopes were compared between the groups. A difference in dimensionality is reflected by a difference in this slope, with a shallower slope indicating that there is a higher number of dimensions with appreciable signal. The slopes of the linear fit did not differ across the age groups (across all stimuli: *F*(1438) = 2.74, *p* = 0.1). This suggests that the stimulus-evoked responses are not inherently of higher dimensionality in the young. For the sex comparison, males had higher overall power (across all stimuli: *F*(1438) = 71.25, *p* = 10^−16^), for all stimuli and not during rest (*t*(44) = 0.9, *p* = 0.3). Here females had a shallower slope than males (across all stimuli: *F*(1438) = 152.12, *p* = 10^−30^). This suggests a greater complexity of responses within females.

### Replication of results

**Figure 9.**
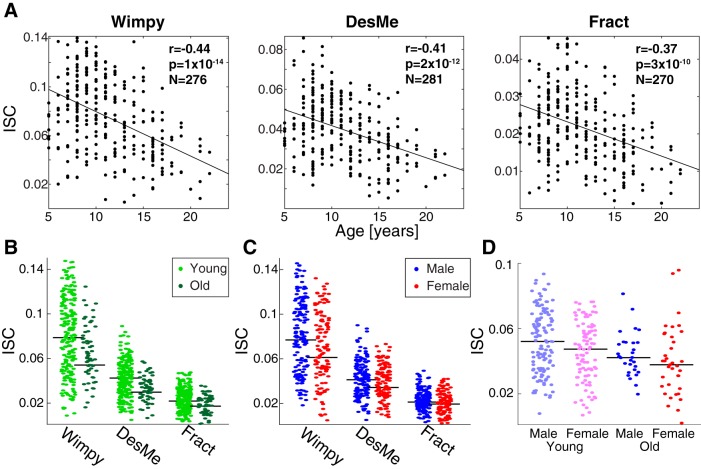
The results from the main study replicated in an independent cohort (*n* = 303). ***A***, ISC decreased with age in the replication cohort. ISC was computed for each individual by correlating responses from individual subjects to those from all other subjects (regardless of age and sex) for that stimulus. Correlation values ranged from *r* = –0.37 to *r* = –0.44. Note that for every stimulus, a different number of subjects was available. ***B***, Across all stimuli, ISC was higher for younger subjects (6–14 yr, light green) than it was for older subjects (15–44 yr, dark green) in the replication cohort. For consistency, the split between the ages was consistent between this study and the main study. ***C***, Across all stimuli, ISC was higher for males (blue) than females (red) in the replication cohort. For both ***B*** and ***C***, ISC was computed separately within each age and sex group. Black lines indicate the median. ***D***, Sex differences in the young disappeared with age in the replication cohort. Young males were more neurally similar to each other than young females, and this sex difference was absent in the older group. Here, ISC was computed within each sex and age group separately and averaged across all stimuli used in the replication cohort. Black lines indicate the median.

To confirm these findings, the results were replicated in an independent cohort (*n* = 303) with a reduced stimulus set: Wimpy, Fract, and DesMe. Replicating the results above, ISC also decreased with age in this cohort (Wimpy: *r* = –0.44, *p* = 10^−14^, *n* = 276; Fract: *r* = –0.37, *p* = 10^−10^, *n* = 270; DesMe: *r* = –0.41, *p* = 10^−12^, *n* = 281; Fig. 9*A*). A two-way ANOVA with age and condition as factors revealed that ISC is modulated by age (*F*(1799) = 35.33, *p* = 10^−9^) and stimulus (*F*(2799) = 272.903, *p* = 10^−91^; Fig. 9*B*). A two-way ANOVA with sex and stimulus as factors revealed that ISC was also significantly modulated by sex (*F*(1823) = 11.12, *p* = 0.0009; Fig. 9*C*) and stimulus (*F*(2823) = 430.95, *p* = 10^−129^). Finally, a two-way ANOVA that divided the data across age and sex groups and averaged ISC across stimuli replicated the main effect of age (*F*(1291) = 17.68, *p* = 10^−5^) and did not find an effect of sex (*F*(1291) = 2.59, *p* = 0.1; Fig. 9*D*). Follow-up analyses that examined a potential sex difference in ISC in each age group revealed that the difference in ISC was present at young ages (*t*(224) = 2.29, *p* = 0.02, 5–14 yr), but not old ages (*t*(67) = 0.59, *p* = 0.6, 15–21 yr). When the median was calculated according to the median of the replication distribution (split at 10/11 years; see Fig. 1B for age distribution), the above results were unchanged. In summary, all results from the main experiment were replicated in this independent cohort.

## Discussion

The present work demonstrated age- and sex-related variability among individuals with respect to their neural responses to complex naturalistic stimuli. Specifically, ISC was significantly correlated with age for both naturalistic videos and artificial visual flashes. Younger subjects (6–14 yr) exhibited less-variable neural responses than older subjects (15–44 yr). A parallel finding revealed that young males exhibited more similar responses to the stimuli than young females, a difference that was present only in the younger cohort. These age and sex effects may result from neural development, consistent with the notion that neural maturation occurs later in males than in females (Lenroot et al., 2007; Marsh et al., 2008; Mous et al., 2017). A quantitative analysis of the spatial distribution of the correlated activity revealed that the observed age and sex differences are largely driven by the same neural components, lending more weight to the idea that the observed differences in age and sex stem from a common developmental feature. Finally, a replication study with 303 participants yielded similar results.

A possible confound for the present results is that the neural correlations found across subjects are due to correlations in overt behaviors such as eye movements. However, it is unlikely that eye movements follow the same developmental trajectory as neural responses, because eye movement trajectories evoked by videos actually become more similar with age (Kirkorian et al., 2012). Thus, although the gaze patterns evoked by videos seem to converge with maturity, potentially driving similar bottom-up neural processes, neural similarity as measured by ISC, decreases with age. This indicates that patterns of neural activity may potentially increase in their diversity with age as top-down factors relating to the interpretation of naturalistic stimuli develop. Even in the condition where subjects were instructed to maintain a fixed gaze position (Flash), ISC decreased with age. Future studies with fine-grained eye-tracking during EEG could more definitively answer this question.

The observed ISC magnitude changes with age and sex may also be partially dependent on evoked response magnitudes, which typically decrease with age. Although the amplitudes of auditory ERPs and their components decline with age (Goodin et al., 1978; Tomé et al., 2015), other components increase with age (Dinteren et al., 2014) or remain stable across development (Kujawa et al., 2013). Although correlation, as measured by ISC, is in principle insensitive to magnitude, it is possible that weaker stimulus-evoked responses in adults may be overpowered by non–stimulus-related neural activity (i.e., “noise”; Hämmerer et al., 2013). In this case, a smaller fraction of the signal would correlate across adults in comparison with children. To control for the effect of age, the magnitude of SSVEPs was regressed from the ISC. The result indicates that SSVEP amplitude cannot explain the age effect, but it may explain the sex effect, indicating that males have stronger evoked responses than females (Figs. 3*B* and 6*B*). However, it is worth noting that ISC and SSVEPs measure very different facets of neural activity. SSVEPs, extracted from early visual processing areas in V1, likely represent low-level visual processes. ISC, on the other hand, may be driven by higher-level cortical areas, since the spatial distributions of the two dominant components (Fig. 7) do not resemble low-level sensory evoked responses. Parallel work indicates that the first component (C1), which captures the majority of the correlated activity, is a supramodal component that is driven by both auditory and visual stimuli (Cohen and Parra, 2016).

It is also possible that ISC decreases with age because adults process the world with more diverse brain activity. In this view, adults have more highly variable stimulus-evoked responses, and their neural activity is therefore less similar across subjects. In this case, it would be likely that the dimensionality of neural responses, a measure of their complexity, increases with age (Anokhin et al., 2000; Mcintosh et al., 2008; Vakorin et al., 2013). There was no clear trend indicative of a difference in the dimensionality between the young and old group. However, it does appear that females have more diverse responses than males, a result that deserves further exploration and could possibly underlie the reduction in ISC in this group.

The present results appear to be consistent with Campbell et al. (2015), who, using fMRI, also found a decrease of ISC with age. However, whereas we study an age range dominated by development and corresponding improvements in cognitive performance (6–44 and 5–23 yr in each cohort), Campbell et al. (2015) examined a range (18–88 years) that exhibited a deterioration in fluid intelligence and reaction time. These measures correlated with a decrease in ISC. Whereas Cantlon and Li (2013) studied a cohort that was more comparable to ours in (4–25 yr), they found that ISC of fMRI was generally higher among adults (above age 18) than it was in children (below age 11). In total, it appears that ISC as assessed by fMRI increases with development and declines in older age, which potentially opposes our result with EEG. These differences may be due to important methodological discrepancies between these studies and ours. To more definitively establish the effect of age on ISC, more work should be done using both fMRI and EEG.

The idea that maturity is marked by variability is not new (Campbell et al., 2015). It aligns with theories from neural systems modeling and human studies (Mcintosh et al., 2008; Vakorin et al., 2011). In these models, moderate amounts of noise or variability facilitate efficient responses in complex environments. Increased variability may be the reason for reduced evoked-response magnitudes, since ERPs are obtained by averaging across many events that are inherently sensitive to signal noise. It is therefore possible that the increased variability of evoked responses across trials with age results in reduced ERP magnitudes.

In the age range examined, neural development is a dynamic process. At the macro level, longitudinal structural neuroimaging shows that cortical thinning occurs from childhood through early adulthood, progressing in a caudal to rostral pattern (Gogtay et al., 2004; Giedd et al., 2015). At the micro level, synaptic pruning and myelination, particularly in the frontal lobe, are ongoing during this period (Rakic et al., 1994; Huttenlocher and Dabholkar, 1997; Cox et al., 2016). From a functional perspective, studies of functional connectivity and task-based fMRI suggest that functional maturation tends to follow a “diffuse to focal” pattern (Durston et al., 2006; Grill-Spector et al., 2008; Fair et al., 2009; Kelly et al., 2009) and may correspond to the extraordinary advances in behavior during childhood (Xiao et al., 2016). Speculatively, the decreased ISC strength in older ages may reflect greater interindividual variability that results from the interplay of structural and functional “streamlining” of neural architecture with distinct life experiences (e.g. cortical thinning, synaptic pruning, and diffuse-to-focal shifts in functional patterns). However, one limitation of the present study is that it is cross-sectional rather than longitudinal, it is therefore difficult to make developmental claims based on the age-based differences demonstrated here (Kraemer et al., 2000).

The age-related effect may also be echoed by the sex difference in neural variability. Longitudinal studies have demonstrated that females mature before males in a range of anatomic measures (Lenroot et al., 2007; Lim et al., 2015). However, differences in developmental trajectories between males and females may be complicated by the fact that the sexes ultimately differ in their mature neuroanatomy (Marsh et al., 2008). Here, sex-related differences in neural variability were seen only among younger subjects, suggesting that this is a development-related difference. Prepubescent and early teenage years are marked by sex differences in behavioral maturity that may not be present in later years (Mous et al., 2017). The difference in neural variability may also be due to pubertal stage, since it is known that females reach pubertal maturity 2–3 yr before males (Sisk and Foster, 2004). However, physiologic pubertal stage was not measured here, and it is therefore not possible to determine whether the sex differences were related to this factor.

Among the different stimuli used, the clips from conventional cinema (Wimpy and DesMe) evoked a higher level of ISC than the educational videos (Arith, Fract, and StudT). The Hollywood clips were rich with scene cuts and dynamic visual cues and are therefore expected to elicit strong levels of ISC (Poulsen et al., 2017). However, previous research has also shown that engagement with narrative stimuli modulates ISC, and it is therefore likely that these Hollywood clips are more effective at engaging attention and thus elicit stronger ISC (Dmochowski et al., 2014; Ki et al., 2016; Cohen et al., 2017). Although the ISC differences between age and sex may be due to each cohort’s average level of attention, no independent measures of engagement or attention were collected. It is therefore not possible to determine whether the present effects are driven by attention or differences in low-level stimulus features. Most of the videos were aimed at younger audiences (i.e., Despicable Me, Diary of a Wimpy Kid), and older subjects may have therefore been less interested in them. However, this was not uniformly the case, for instance, the video about Fractals (Fract) may have been equally interesting to both children and adults, whereas the Flash stimulus may be equally boring for all ages. Thus, these two stimuli provided an important control for attentional effects on the age-related differences in ISC. Future work may benefit from looking at objective measures of engagement (Cohen et al., 2017) in the different cohorts studied here. An understanding of such factors, and their impact on behavior, may be of relevance to models of media-based addiction (e.g., Internet addiction, pornography addiction), as well as commercial neuroscience enterprises. Regardless, it is of note that the age effect seen for the naturalistic videos was echoed in the SSVEP condition. Because this stimulus should be equally (un)engaging for all ages, this favors an interpretation based on neural maturation rather than attention.

Future work should recruit a larger sample of subjects older than 15 yr to determine whether the age-related decline in ISC observed in later teenage years continues in adulthood, or might even reverse in older age (Grady, 2012; Campbell et al., 2015). Future studies with clinical cohorts could explore the potential link between ISC and behavioral markers of neural development. It is possible that neural variability is not only a marker of maturity, but also a marker of neuropsychiatric disorders (Dinstein et al., 2015). The methods used here provide a novel way of assessing such markers under complex, naturalistic conditions.

Overall, the current results regarding intersubject correlation in children and adults are interpreted in the context of neural maturation. Although males are delayed in the development of the neural variability that appears to be a mark of maturity, the data presented here indicate that with normal development they are no different from females as adults. Thus, with maturity, neural function becomes more variable.

## Synthesis

Reviewing Editor: Daniela Schiller, Mount Sinai School of Medicine Decisions are customarily a result of the Reviewing Editor and the peer reviewers coming together and discussing their recommendations until a consensus is reached. When revisions are invited, a fact-based synthesis statement explaining their decision and outlining what is needed to prepare a revision will be listed below. The following reviewer(s) agreed to reveal their identity: Christine Tipper, Janice Chen.

This article assesses inter-subject correlation of EEG responses as participants watch naturalistic video stimuli, with a focus on how inter-subject correlation varies with age. The authors analyze data from two large public datasets (N=114 and N=303) with a wide age range; three of the videos are common across the two datasets. The question of how inter-subject variability may change with age is interesting, particularly in the context of naturalistic stimuli, as these conditions approximate real life scenarios much more closely than typical EEG studies. Even beyond mere ecological validity, these videos likely drive complex responses in brain regions that are not normally elicited in EEG studies that use simpler, randomized stimuli.

The overall finding of the paper is that inter-subject correlation decreases with age (or conversely, that inter-subject variability increases with age). The presented results quite clearly support this idea, as can be seen from the strong negative correlations across all videos in Figure 3, and the separation between Young and Old groups in Figure 4a.

While this paper constitutes a compelling report, one major concern is that the conclusions drawn are too broad given the specifics of the experiment. As described below, there are plausible factors potentially influencing the age-variability anticorrelation, as well as possible contradictions with the existing literature, that are not sufficiently discussed. At minimum, these items should be highlighted in the introduction and discussion, and the overall claims be constrained; alternatively (and ideally), these concerns could be addressed with additional data. Below is a list of overall major concerns, followed by line-by-line comments, including additional more minor issues.

Major comments:

1. Unusually low ISC across the sample:

The authors cite a few papers that use the same ISC methods as the current study (line 176). In these papers, ISC values are higher than those in the current study. In Cohen et al. (2016) eNeuro, adults (age 25 +/- 6 years) watched short videos (161 +/- 44s); ISC in this study was reported to be around 0.1 on average, with a standard deviation of around 0.02 (eyeballing Figure 3 AV condition). A similar range of ISC is reported in Ki et al. (2016) J Neurosci: mean ISC = around 0.1 for an attended audiovisual movie (Figure 2 condition BYD), with a total range of around 0.09 to 0.15. In this case, the movie was a 6-minute Hitchcock clip, and the participant age range was 18-30. By contrast, the current study reports mean ISC values of around 0.01-0.03, as seen in Figures 3 and 4 (perhaps as high as 0.05 in Figure 4). In fact, Figure 4 shows that the mean ISC for Old-group participants (ages 15-44) is below 0.02 in all but one of the 5 audiovisual movies. In short, the Old-group age range corresponds to the age range used in the above two studies, but the ISC is much lower here. Only in the highest-scoring movie (“Wimpy”) do the Young-group participants come close to the reported ISC of the other papers (near 0.1). For reference, ISC values in fMRI are higher in general then those reported in EEG; for example, see Lahnakoski et al. (2017) J Neurosci, Figure 4, ISC ranging from 0.03 to over 0.5. It is not entirely fair to compare fMRI and EEG ISC, for many reasons, but the method used in Lahnakoski et al. (pairwise correlation) is, it seems, roughly analogous to the method employed in the current paper (sum of 3 top components from correlated component analysis, also pairwise across subjects). This is simply to make the point that ISC values (which are Pearson correlation values, essentially), across multiple imaging methods, are usually considerably higher than what is reported in the current paper.

One possible explanation for the low ISC in this paper is that the data are of poor quality. This in itself wouldn't necessarily introduce bias or artificially produce the results that 1) variability increases with age, 2) males are more variable than females, and 3) the gender difference disappears with time. It's possible that the data in young and old subjects vary in quality in just the right pattern to produce these results (for example, if the data became increasingly noisy with increasing age), but it is unclear how we would determine that. Overall, the low ISC is cause for concern about the data quality, and undermines the soundness of the results.

2. Attention as an alternative explanation:

Another explanation, which the authors touch on briefly in the Discussion, is that ISC is especially low among adult participants because all of the videos are aimed at a younger (child) audience (line 466). This seems rather plausible. As shown in Ki et al. (2016), lack of attention to an audiovisual movie can dramatically reduce EEG ISC. In other words, ISC decreases with age because subjects' interest in the videos decreases with age. If this is indeed the real explanation for the low ISC in the Older group, then it would be an incorrect conclusion that age and variability are anticorrelated beyond this dataset. There may not be a way to rule this out with the available data.

3. Coherence with the extant literature:

As described in the Introduction, there are a few other studies out there which show that inter-subject behavioral variability decreases with age; and at least one fMRI study showing that neural responses are less variable in adults than children (counter to the current study), even when watching a video program designed for children (Cantlon & Li, 2013; although one region is found showing the opposite pattern). While another study (Campbell et al.) is described which shows an increase in variability with age (congruent with the current study), the age range was quite different from the current study: 18-88 in Campbell et al., which would fully fall into the Old-group condition used in this paper (ages 15-44) and thus does not serve as a good comparison for the current findings. Overall, we would expect that readers familiar with ISC from the fMRI literature would find the notion that ISC decreases with age to be pretty surprising and counterintuitive. Going against the extant literature is not necessarily a detriment per se -- in fact it would be extremely interesting if EEG and fMRI produced opposite patterns of age vs. ISC -- but the apparent contradictions need to be discussed, and potential reasons suggested.

4. Relevance to other measures of neural variability, and possible missing Results:

In the Introduction, the authors open by describing the “seemingly paradoxical increase in the variability of neural responses” and “an increase in the complexity of neural responses” with age, citing a body of work on EEG variability from researchers including Grady, Dinstein, and McIntosh. This is intended as background or setup for the age-variability correlation results in the current paper. However, it is not clear how solid the analogy is. McIntosh 2008, for example, states that, “our focus was not on variability of the signal across individuals within a group (interindividual), but rather the single-trial variability within an individual (intraindividual)”. This seems pretty different from (explicitly contrasted with, in fact) the measures used in the current paper; this should be clarified in the writing, as it could be confusing for readers who incorrectly infer that McIntosh's neural variability is the same measure as the ISC variability referred to throughout the current paper. Additionally, could the authors please elaborate on what they think the relationship is between ISC and McIntosh's neural “variability”? This is touched on at line 410, but the message of this paragraph isn't clear. A statement is made here that sounds like a result (”A clear trend, indicative of a difference in the dimensionality between either the young and old group, or males and females, was not apparent in these data”), but we could not find any analysis in the paper relating to “dimensionality”. Is there a section missing from the Results? (To be clear, the term “variability” in comments 1-3, refers to inter-subject variability).

In summary of major issues: At minimum we ask that suitable attention be given to the above points in the Introduction and Discussion, and that the overall claims be constrained accordingly. Alternatively, these concerns could be addressed with additional analyses, e.g., showing the age-ISC anticorrelation in videos that are not aimed at a child audience, and preferably with ISC values in a range more congruent with previous literature. As it stands, it is premature to conclude from the current data/analyses that inter-subject correlation decreases with age.

Specific (major and minor) comments:

Title “Age and sex modulate”... This title infers causality in the observed age and sex results, which were all correlational in nature.

Line 9 The data show that young males were shown to have LESS variable responses, not MORE.

Lines 14-19 It seems that some of the content included in the significance statement is actually novel info about results, and thus belongs in the abstract. The significance statement should be used to comment on the importance of the findings.

Line 25 “variability neural responses”... Be more specific here. What kind of neural responses? What kind of variability?

Line 25 “More variable”... please clarify what this means (computationally and/or quantitatively)

Lines 28-29 What do you mean by the “complexity of neural responses”?

Line 32 What do you mean by “neural maturity”? This concept is vague and ambiguous.

Line 36 “more variable in children”... How? Over time? Between individuals? More precision in these key concepts would help tremendously.

Line 44 “more variable gaze patterns”... As indicated by what? Variable how? What aspects of gaze?

Lines 46-47 “more similarly”... More than what? Is what you're trying to say here is that in adults ISC is greater in parietal and frontal regions? Or that there is greater connectivity between parietal and frontal regions. Please clarify this statement.

Lines 47-49 This sentence seems to contradict the evidence regarding GREATER variability in kids cited immediately above. Specifcially, the authors are pointing out that Cantlon & Li show that adults have more “broadly similar” neural responses than children, ie higher adult-group ISC than child-group ISC throughout most of the brain, but in one region (STC) the opposite pattern was found. In all cases in this paragraph, “similar” and “variable” refer to “within-group across-subject similarity and “within-group across-subject variability”. This type of language is relatively common in ISC papers, but the authors should be more clear with their phrasing. This also relates to comment #4 above, as in paragraph 1 it appears they are talking about inter-trial “variability””, but continue to use the same term when switching to inter-subject “variability” in paragraph 2. That needs to be fixed.

Line 49 “Trajectory”... This is another unexplained/uncontextualized concept. How can the authors talk about trajectory when there has been no mention/observation of longitudinal changes.

Lines 52-56 It seems as if the research question could be stated more clearly, and earlier in the introduction as well. As it currently reads, there is no strong apparent direction. It would help if the authors made their purpose explicit at the outset, and then describe the relevance of previous results to their question.

Line 58 “females have more variable responses”... This matches the data, but not the abstract.

Lines 85-86 “ranged in engagement level”... What does this mean? How is engagement measured? What exactly counts as engagement? How is this statement supported? With what metrics? How do they conclude that the educational videos were “weakly engaging”?

Line 120 “three condition” ... Should read “three conditions”.

Lines 145-146 “as described previously”... This is such a key concept/tool in this study; it really deserves greater explanation/computational description HERE (which might forestall the reviewer's questions about Lines 147, 160, 163, 167-169, and 170-178).

Line 147 “level of correlation in the EEG” ... This is too vague. Correlation in time? Between channels? Correlation of what? Voltage potentials? How is this computed?

Line 156 The concept of a priori does not apply here. In fact, a priori refers to decisions/hypotheses/conclusions made based in reason or logic, in absence of actual observations. So, it's actually kind of the opposite of data-driven.

Line 156 “data-driven both spatially and temporally”... Not clear what this means, or what the logic is here. How does not requiring event markers influence this?

Line 160 “maximize the correlation”... Of what? On a sensor-by-sensor basis? Or does this project take into account the full spatial distribution of electrodes? This should be specified.

Line 163 “most of the correlation” ... Again, this needs to be spelled out in terms of computation. How is this being quantified? How much correlation was there between subjects? What data was input into this analysis, exactly?

Lines 167-169 Please explain how computing ISC separately for different subsets of data allows for observing differences in spatial patterns?

Lines 170-178 Please include more detail about ISC computation in this manuscript. As it stands, it is too vague to get a good understanding of the data and results.

Line 191 How was this power calculation implemented in Matlab? Were any plugins/other (standard) software used? Power of what, exactly? The authors might be referring to power in the 25Hz band, but this statement comes before the description of the frequency-specific power analysis.

Line 194 “25Hz range”... What was the bandwidth?

Line 207 “Ranged in engagement level”... How can stimuli themselves range in “engagement”? Isn't engagement defined based on the viewer's experience, as opposed to the content of the videos? Is there any way the authors can support this subjective statement with data? If not, perhaps they should drop the concept of “engagement” as being a part of the independent (manipulated) variable and just report the differences in neural variability between stimuli. The authors could then interpret/discuss the results as indicative of greater or lesser engagement without raising the specter of circularity caused by using “engagement” as both an independent variable and an interpretation.

Lines 210-211 “ISC is a stimulus-driven measure of attention”... What does this mean? How so? Please explain this relationship. Why bring in the idea of attention at all? Why not just stick to an examination of “stimulus-driven neural activity”? Presumably, the authors are referring to the results of the cited paper, Ki et al 2006. They could rephrase the sentence to make that clear.

Lines 212-219 Why were two separate one-way ANOVAs performed here? It is not clear how the second differs from the first, except for having dropped the Rest condition. Why not just compute the Tukey's tests on the first ANOVA?

Line 213 What do these ISC values reflect? These seem like VERY low numbers. Are they comparable to Pearson's coefficients? If so, can these tiny numbers really be considered meaningful/significant correlations? The authors report that ISC for Rest was not significant from zero (with a one-sample t-test). What about the other conditions? Were they tested as well?

Line 230 we recommend that the scales for the different conditions be equivalent for ease of comparison.

Lines 230-234 this caption needs to be clarified. It seems the authors are conflating ISC with age-ISC correlations. The last sentence of the caption refers to age-ISC correlations, but it immediately follows a description of ISC computation, so it's confusing.

Line 248 this median split analysis is quite important to include, the reason presumably being that the correlated component analysis *could* have produced spatial weight maps that were different (and perhaps more effective) for children than adults, e.g., if there was a lot of variability across age in terms of where electrodes sit on the scalp relative to the brain. Thus, they needed to show that ISC differs between groups even when fitting the age groups separately. Ideally, they could have split the sample even further, as 15-44 is a pretty large age range. Nonetheless, the authors should explain their rationale better: why the authors conducted this as a second analysis. If age sampling was considered a potential problem, why not just conduct this median split analysis in the first place? And it is also unclear why ISC had to be recomputed after regrouping participants, why not just re-average ISC values? This should be made explicit.

Line 255 There were many age-ISC correlations computed between the two analyses. Did the authors make any attempt to control for multiple comparisons? This should be addressed.

Line 258 “Across all stimuli”... Figure 4 seems to align with the stimulus-specific analysis, but the caption seems to indicate otherwise. Do these data show separate points for each stimulus, for each subject? Wouldn't this invalidly inflate the degrees of freedom for the correlation analysis? Or do these data reflect ISC averaged over stimuli? Or do they reflect a separate ISC computation distinct from that used in the analysis described above? More specifically, what is meant by inflating the df was that if there are 5 data points for each participant in a correlation analysis, without including “subject” as a random effect factor, each stimulus will be treated as a unique independent data point (which they are not), and this will increase Type I error rate. What the authors should ensure is that even if they compute ISC separately for each stimulus, that in their age-ISC and sex-ISC correlation analyses, ISC should be averaged across stimulus types to create a single data point for each participant.

Lines 265-275 Why do the authors compute 2 separate two-way ANOVAs as opposed to a single 3-way ANOVA, with stimulus, age, and sex as factors. That way, the authors could directly test for age x sex interactions, which seems critical, as this interaction constitutes an important part of their findings and conclusions.

Line 272 It is not clear how averaging “across all stimuli available” would have any effect on sample size. Also, it is not apparent why ISC needed to be recomputed again.

Line 279 “disappear with age”... This is not tested, strictly speaking. There are no longitudinal observations.

Line 283 “Age's effect”... Would read more easily as “the effect of age on...”

Line 290 “and two-way ANOVA”... Should read “and a two-way ANOVA”.

Line 300 “evoked response strength”... Please be clearer about what “response” you are referring to. Presumably, it's 25 Hz power, but this should be reiterated here.

Line 323 “forward model”... please define what this is, since eNeuro has a general audience.

Line 324 “these component topographies were very similar”... How was this measured/tested? It is unclear whether a simple visual inspection is adequate here. It does appear that there may well be meaningful differences in topographies. C3, for example, is quite different between age/sex groups. And for C1, it seems like the frontal positivity becomes more anterior and focal with age, and in females. For C2, it looks like the lateral positivity is smaller/more focal in males, and perhaps larger with age. Could the authors please conduct some kind of permutation test to quantify these component topographies?

Line 325 “Figure 7”... Should read “Figure 8”.

Line 327 Why would the authors use the numbers based on Figure 3 and average across stimuli rather than using the ISCs from the analysis represented by Figure 8? The authors should find a way to compute ISCs once and conduct all of their stats based on this single computation. If this is impossible, the rationale needs to be better explained, and there needs to be far more care to clarify exactly what numbers are being tested in each analysis and why.

Line 346 Was ISC computed here for ALL stimuli together, or for each stimulus separately and then averaged?

Lines 378-379 “observed age and sex differences are largely driven by the same neural components”... How do the authors arrive at this conclusion? It is not valid to say that since the component topographies looked superficially similar upon visual inspection, that the age and sex DIFFERENCES must be driven by the same mechanisms. Please clarify the logic here.

Line 413 “dimensionality of neural responses, a measure of their complexity”... These are two entirely new concepts in the manuscript, and should be explained more fully. What “dimensions” can neural responses have, exactly? And what relates this to complexity?

Lines 415-416 “was not apparent in these data”... How was this assessed? Do the authors have any quantitative backup to this claim?

Lines 417-424 This section doesn't seem pertinent to the results or conclusions of the study, and also reads as unfocussed. Consider dropping this paragraph, or making its purpose more evident.

Lines 430-431 It is not clear how differences in ERPs are relevant to this study.
